# Microalgae-based biodiesel: integrating AI, CRISPR and nanotechnology for sustainable biofuel development

**DOI:** 10.1042/ETLS20240004

**Published:** 2025-09-22

**Authors:** Fariha Kanwal, Ambreen Aslam, Angel A. J. Torriero

**Affiliations:** 1 Department of Environmental Science, Lahore College for Women University, Pakistan; 2 Department of Environmental Science, University of Lahore, Pakistan; 3 School of Life and Environmental Sciences, Faculty of Science, Engineering & Built Environment, Deakin University, Burwood, VIC, 3125, Australia

**Keywords:** biodiesel, harvesting, microalgae, sustainability, transesterification

## Abstract

Microalgae are a promising feedstock for biodiesel due to their rapid growth, high lipid content and ability to use non-arable land and wastewater. This review synthesises recent advances in artificial intelligence (AI)-driven strain optimisation, engineering, nanotechnology-assisted processing, and life cycle and technoeconomic insights to evaluate pathways for industrialisation. Over the past decade (2015–2024), genetic engineering and, more recently, AI-guided strain selection have improved lipid productivity by up to 40%. Cultivation advances, including hybrid photobioreactor–open pond systems and precision pH/CO_2_ control, have enhanced biomass yields while reducing costs. Innovation in lipid extraction, such as supercritical CO_2_ and microwave-assisted methods, now achieves >90% yields with lower toxicity, while magnetic nanoparticle-assisted harvesting and electroflocculation have reduced energy inputs by 20–30%. Life cycle analyses (net energy ratio ~2.5) and integration of high-value co-products (e.g. pigments and proteins) underscore the need to align biological innovations with techno-economic feasibility. This review uniquely integrates advances in AI, CRISPR and nanotechnology with life cycle and techno-economic perspectives, providing a comprehensive framework that links laboratory-scale innovation to industrial feasibility and positions microalgal biodiesel as a viable contributor to global decarbonisation strategies.

## Introduction

Over the past two decades, global energy strategies have increasingly shifted toward renewable technologies to mitigate climate change, improve energy security and stabilise economies [1]. The extensive use of fossil fuels has caused severe environmental consequences, including carbon dioxide emissions, biodiversity loss and pollution [2–4].

Recent advances in renewable energy technologies – such as solar photovoltaics, wind and biofuels – have reduced renewable energy costs by 50–80% since 2010, enabling decentralised power systems that expand energy access in marginalised regions [5–8]. This transition has reshaped geopolitical influence, with nations leading in renewables gaining strategic leverage over fossil fuel-exporting economies.

Within this context, biodiesel has emerged as a prominent renewable transport fuel, contributing over 50% of global biofuel output and serving as a carbon-neutral alternative to conventional diesel [9,10]. Produced through transesterification of long-chain fatty acid methyl esters (FAMEs), biodiesel aligns with existing engine infrastructure and offers immediate decarbonisation potential.

**Figure 1:**
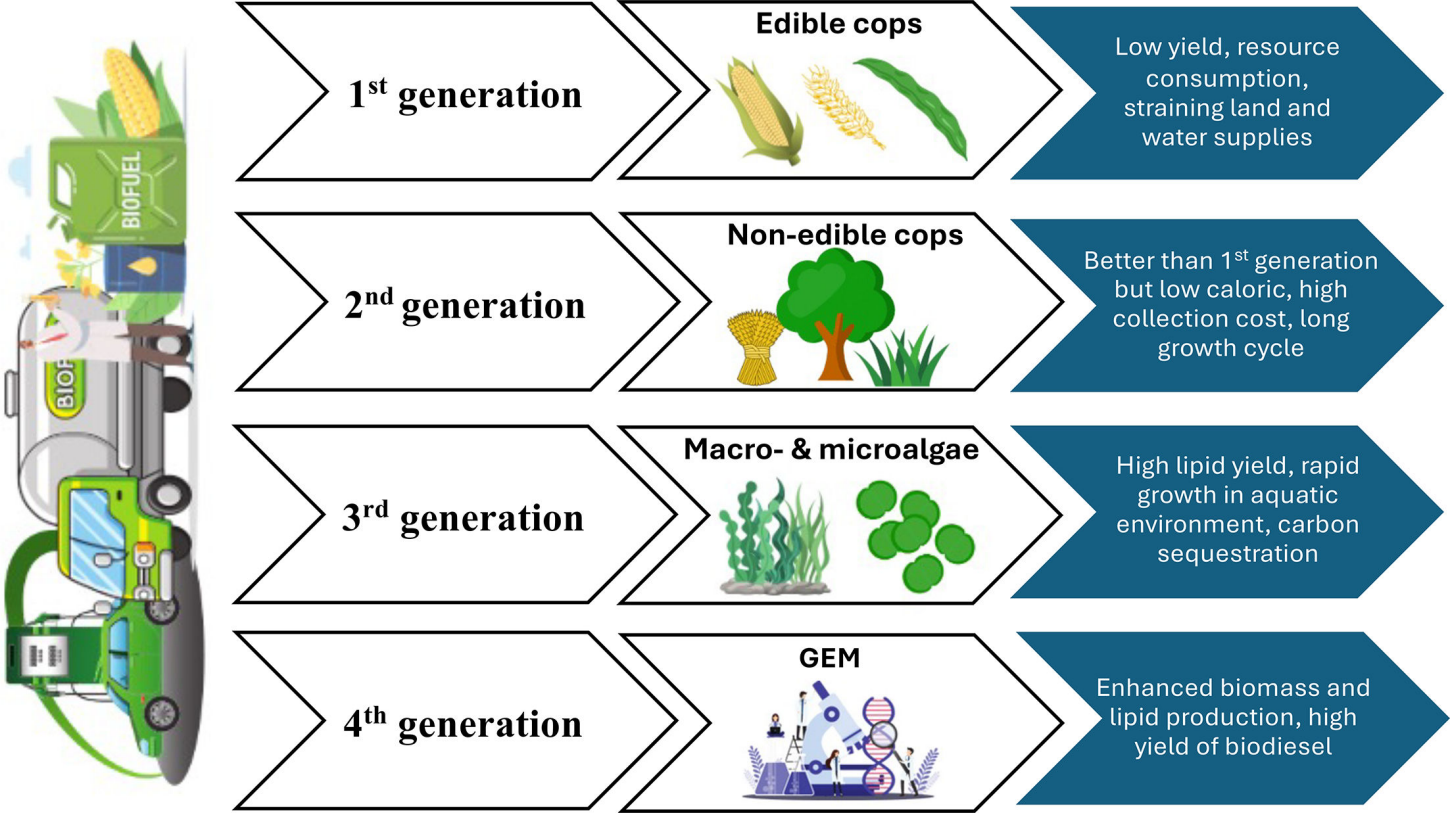
Evolution of biodiesel feedstocks across four generations, illustrating the transition from edible crops to genetically engineered microalgae (GEM) and the corresponding improvements in efficiency. Created by the authors.

Feedstock development has progressed through four generations (Figure 1): first-generation edible crops, second-generation non-edible crops, third-generation microalgae, and now fourth-generation genetically engineered microalgae using tools such as CRISPR-Cas9 [11]. First-generation biodiesel faced ethical challenges due to food competition, while second-generation non-edible feedstocks were constrained by high collection costs and limited caloric efficiency. Third-generation microalgae offer lipid productivities exceeding 40% of dry weight, rapid doubling times and the ability to grow on wastewater or saline resources [10]. Fourth-generation systems build upon these traits by incorporating genetic engineering and synthetic biology to enhance stress tolerance and compatibility with integrated biorefineries, creating scalable and resource-efficient pathways.

This review synthesises recent technological breakthroughs – including artificial intelligence (AI)-guided strain optimisation, CRISPR-based engineering, hybrid photobioreactor (PBR)–open pond cultivation, magnetic nanoparticle-assisted harvesting, supercritical CO₂ extraction and nanocatalyst-assisted transesterification – and evaluates their implications for scalability, sustainability and integration into circular bioeconomy models. Unlike recent reviews in Fuel, Algal Research and Renewable Energy, which have emphasised mainly cultivation and conventional processing, this article integrates these advances with life cycle (net energy ratio [NER]) metrics and techno-economic feasibility, providing a forward-looking perspective that links laboratory innovation to industrial application.

Biodiesel production from microalgae offers a sustainable approach by reusing and integrating carbon resources from the environment, contributing to the global goal of reducing petroleum use and environmental pollution [9,12–15]. Microalgae, more efficient than macroalgae due to their higher lipid productivity and easier cultivation, are rich in compounds like lipids, proteins and carbohydrates. In addition to environmental benefits, economic sustainability remains critical for biofuel viability. Profitability and favourable investment conditions influence technology adoption and scale-up, as highlighted by analyses of investment decision-making in emerging industries [16,17]. These factors reinforce the importance of techno-economic feasibility, life cycle metrics (NER ~2.5), and co-product valorisation in aligning microalgal biodiesel development with industrial and investor expectations.

## Microalgae as a feedstock for biodiesel production

Microalgae are photosynthetic organisms thriving in aquatic environments, including marine and freshwater habitats. These organisms, which can be unicellular or multicellular, exhibit remarkable adaptability, thriving under diverse conditions and displaying various metabolic modes such as autotrophy, heterotrophy, mixotrophy and photoheterotrophy [18,19]. Importantly, their cultivation does not compete with food production as they can grow in freshwater, saltwater, saline water and even wastewater.

**Figure 2:**
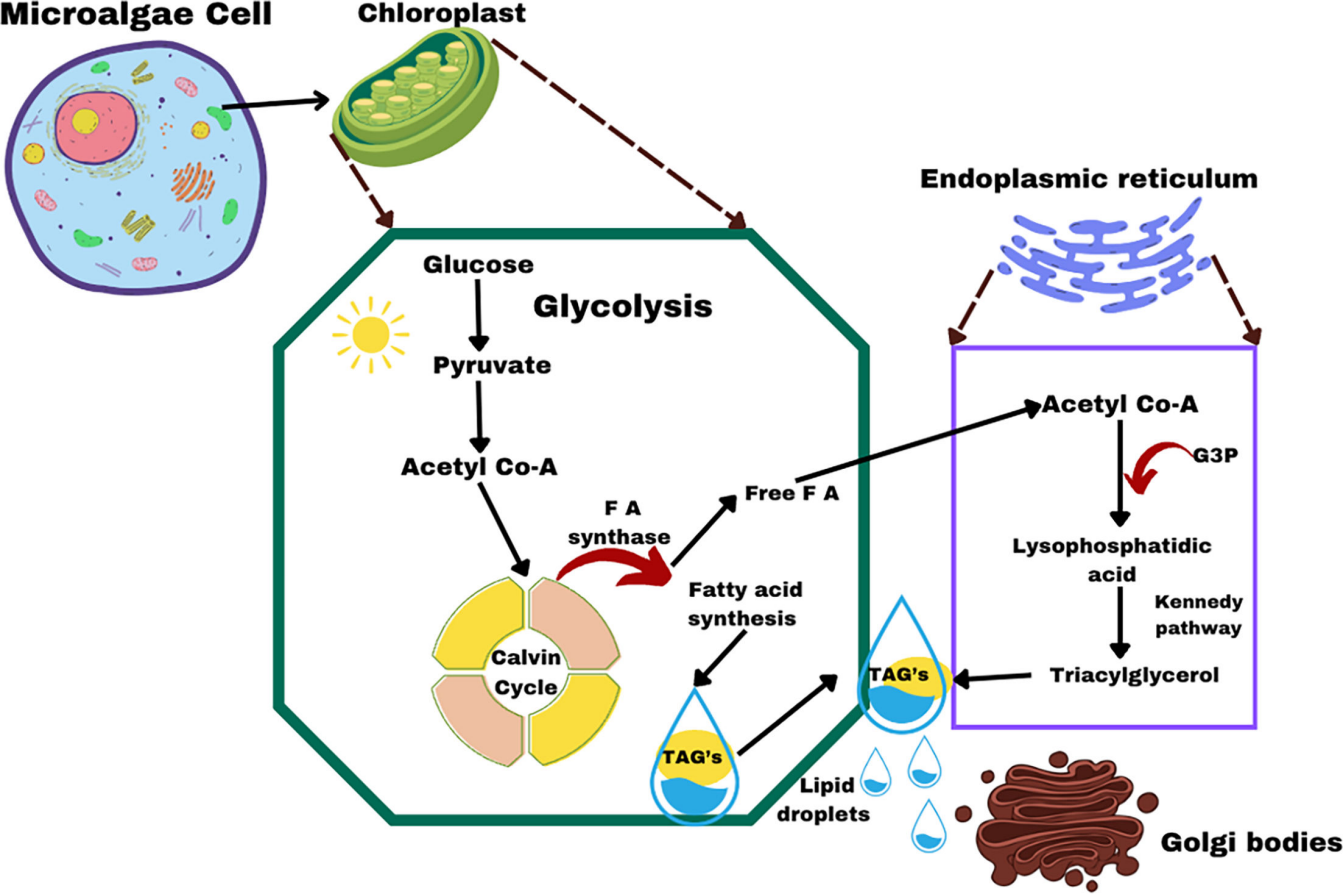
Schematic overview of lipid synthesis in microalgae: glucose undergoes glycolysis to form acetyl-CoA, which is converted into fatty acids in the chloroplast and subsequently assembled into triacylglycerols (TAGs) in the endoplasmic reticulum. These pathways underpin microalgal biodiesel production. Created by the authors.

Microalgae are primarily composed of lipids, carbohydrates and biomass, with their lipid content making them highly suitable for biodiesel production. Lipids, which are biological molecules dissolvable in organic solvents, are categorised into polar and non-polar lipids, with fatty acids serving as their key components. Fatty acids vary in chain length, saturation and structure, forming either neutral lipids (e.g. triacylglycerols) or polar lipids (e.g. phospholipids) [20–24]. The lipid synthesis process in microalgae occurs primarily in the chloroplast, where glucose undergoes glycolysis to generate acetyl-CoA, a precursor for lipid production, as depicted in Figure 2 [10, 11 and 21].

Certain microalgae species, such as *Chlamydomonas reinhardtii* (21% lipid content), *Spirulina platensis* (8% lipid content) and *Chlorella* species (19% lipid content), have garnered significant research attention due to their potential as biodiesel feedstocks [25,26]. Oleaginous microalgae, characterised by their high oil yields, represent particularly promising candidates. Detailed lipid content and fatty acid profiles for various algal species are presented in Table 1, which underscores the variability in lipid productivity across species [25,26].

**Table 1:** Lipid content and fatty acid profiles of selected microalgal species.

Sr. no	Mode	Species	Lipid content % (dry cell weight)	FA profile	Ref.
**1**	Autotrophic	*Nannochloropsis sp*.	12–53	C16:0;C16:1;C18:1;C18:2;C20:4;C20:5	[27]
**2**	Autotrophic	*Dunaliella sp*.	17.5–67	C16:0;C16:1;C18:0;C18:1;C18:2;C18:3	[28]
**3**	Autotrophic	*Phaeodactylum tricornutum*	20–40	C16:0;C16:1;C18:0;C18:1;C18:2;C20:5	[29]
**4**	Heterotrophic	*Crypthecodinium cohnii*	51	C16:0;C18:1;C18:2;C18:3;C22:6	[30]
**5**	Mixotrophic	*Dunaliella tertiolecta*	17.5–67	C16:0;C18:1;C18:2;C18:3	[30]
**6**	Autotrophic	*Chlamydomonas sp*.	20	C16:0;C18:0;C18:1;C18:2;C18:3	[29]
**7**	Autotrophic	*Chlorella sp*.	40	C16:0;C18:0;C18:1;C18:2	[31]
**8**	Autotrophic	*Desmodesmus sp*. EJ8-10.	19.4–28	C16:0;C18:1;C18:2;C18:3	[9]
**9**	Autotrophic	*Scenedesmus abundans*	17.3–32.8	C16:0;C18:0;C18:1;C18:2	[32]
**10**	Mixotrophic	*Euglena gracilis*	24.81	C14:0;C16:0;C18:0;C18:1;C18:2	[33]
**11**	Autotrophic	*Botryococcus braunii*	35.9–62.7	C16:0;C18:1;C18:2	[34]
**12**	Autotrophic	*Nannochloropsis oceanica*	49.0–53.2	C16:0;C18:1;C20:5;C22:6	[35]
**13**	Autotrophic	*Boekelovia hooglandii*	59	C16:0;C18:0;C18:1;C18:2;C18:3	[36]
**14**	Heterotrophic	*Schizochytrium limacinum*	40–45	C16:0;C18:1;C20:5;C22:6	[20]
**15**	Autotrophic	*Isochrysis galbana*	30–35	C16:0;C18:1;C20:5;C22:6	[37]
**16**	Autotrophic	*Coelastrella sp*.	44–46	C16:0;C18:0;C18:1;C18:2	[38]
**17**	Autotrophic	*Verrucodesmus verrucosus*	43–44	C16:0;C18:0;C18:1;C18:2;C18:3	[38]

Efficient cultivation is critical for maximising lipid production and biomass yield. Three primary systems dominate microalgae cultivation: open raceway ponds, closed PBRs and hybrid systems. Open systems, while economical, are vulnerable to contamination, whereas PBRs offer controlled conditions for high-quality biomass. Hybrid systems leverage the strengths of both, initially cultivating microalgae in PBRs under optimal conditions, followed by nutrient-depleted open systems to enhance lipid accumulation [25,34,39]. Environmental factors significantly influence lipid synthesis, including light intensity, temperature and nutrient availability. Additionally, salinity stress and pH optimisation have been shown to further enhance lipid accumulation [18,40–42].

Harvesting and dewatering microalgae biomass involves thickening to increase solid concentration and dewatering for separation. Techniques include physical methods like flotation, filtration and centrifugation and chemical, biological, magnetic and electrochemical methods. Emerging approaches, such as magnetic harvesting using nanoparticles and electroflocculation, show the capacity to reduce energy costs and environmental impact [43–45].

Lipid extraction is a pivotal step in biodiesel production, employing hexane, chloroform and methanol solvents. Advanced techniques, including supercritical CO₂ extraction, provide high yields with minimal toxicity but remain cost-prohibitive due to the high pressures involved [46–49]. These processes, alongside ongoing innovations in cultivation and harvesting, position microalgae as a viable and sustainable feedstock for biodiesel production.

## Biodiesel production processes

**Figure 3:**
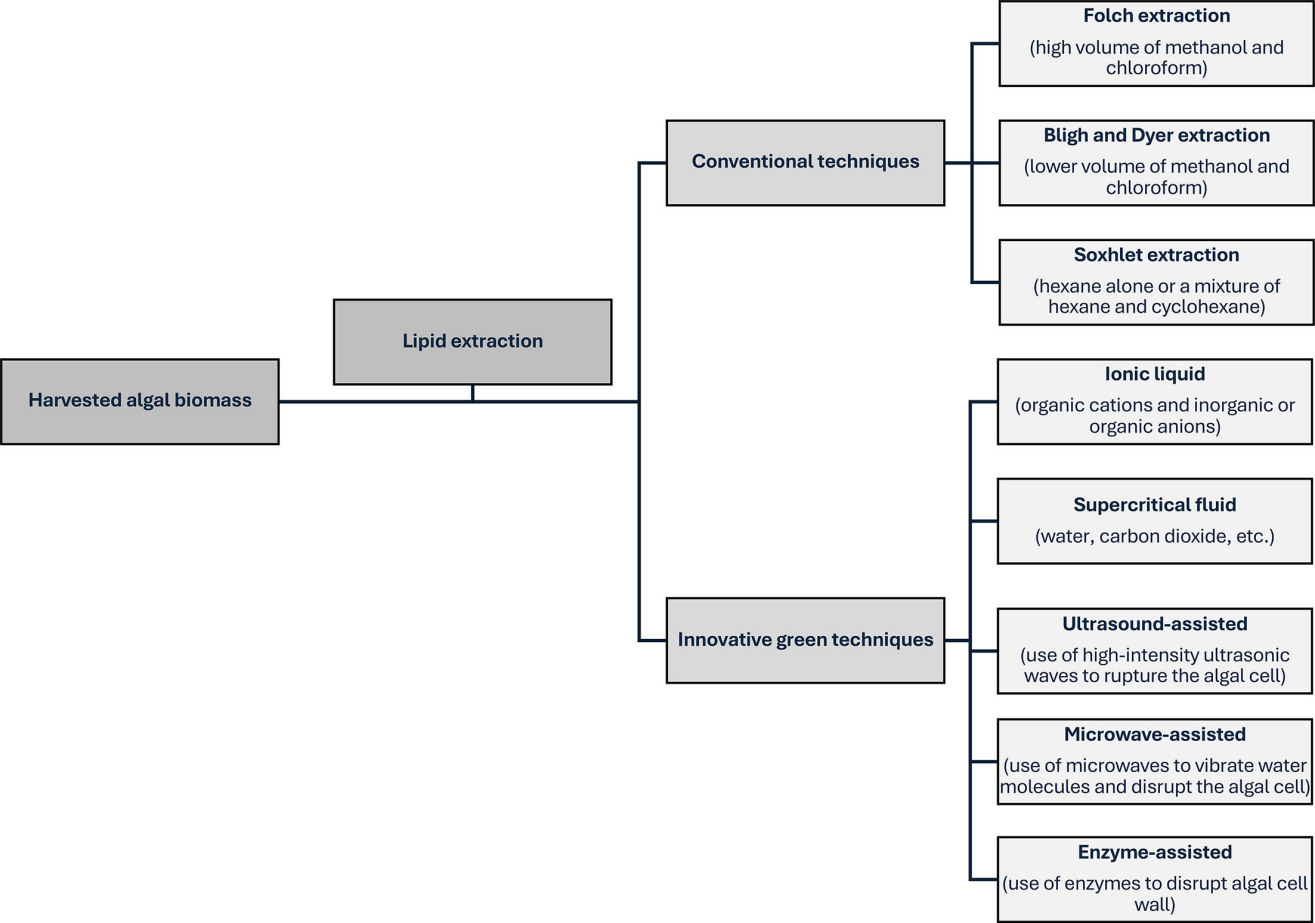
Lipid extraction techniques from harvested algal biomass, comparing conventional methods (e.g. Folch, Bligh and Dyer and Soxhlet) with eco-friendly innovations like supercritical fluids, ionic liquids, ultrasound, microwaves and enzyme-assisted processes.

Microalgae oil is a promising feedstock for biodiesel due to its sustainability and high lipid content. While transesterification remains the predominant conversion route for biodiesel, alternative methods such as hydroprocessing and pyrolysis are also under investigation. However, these pathways are less mature and not yet widely applied to microalgal feedstocks [50]. Nonetheless, microalgae oil’s high viscosity and low volatility present challenges for engine performance. These issues are addressed through transesterification, a chemical process that converts triglycerides into biodiesel, specifically FAMEs, and glycerol using alcohol (typically methanol) and a catalyst [51]. The lipid extraction techniques preceding this process are illustrated in Figure 3.

### Transesterification methods

Two primary approaches to transesterification are non-catalytic and catalytic methods, each offering distinct advantages and challenges. Non-catalytic transesterification, or supercritical transesterification, operates under extreme temperature and pressure conditions and offers rapid reaction times, high yields and simplified purification. This method tolerates diverse feedstocks but is energy-intensive and requires a higher oil-to-alcohol molar ratio [25,34,52].

**Figure 4:**
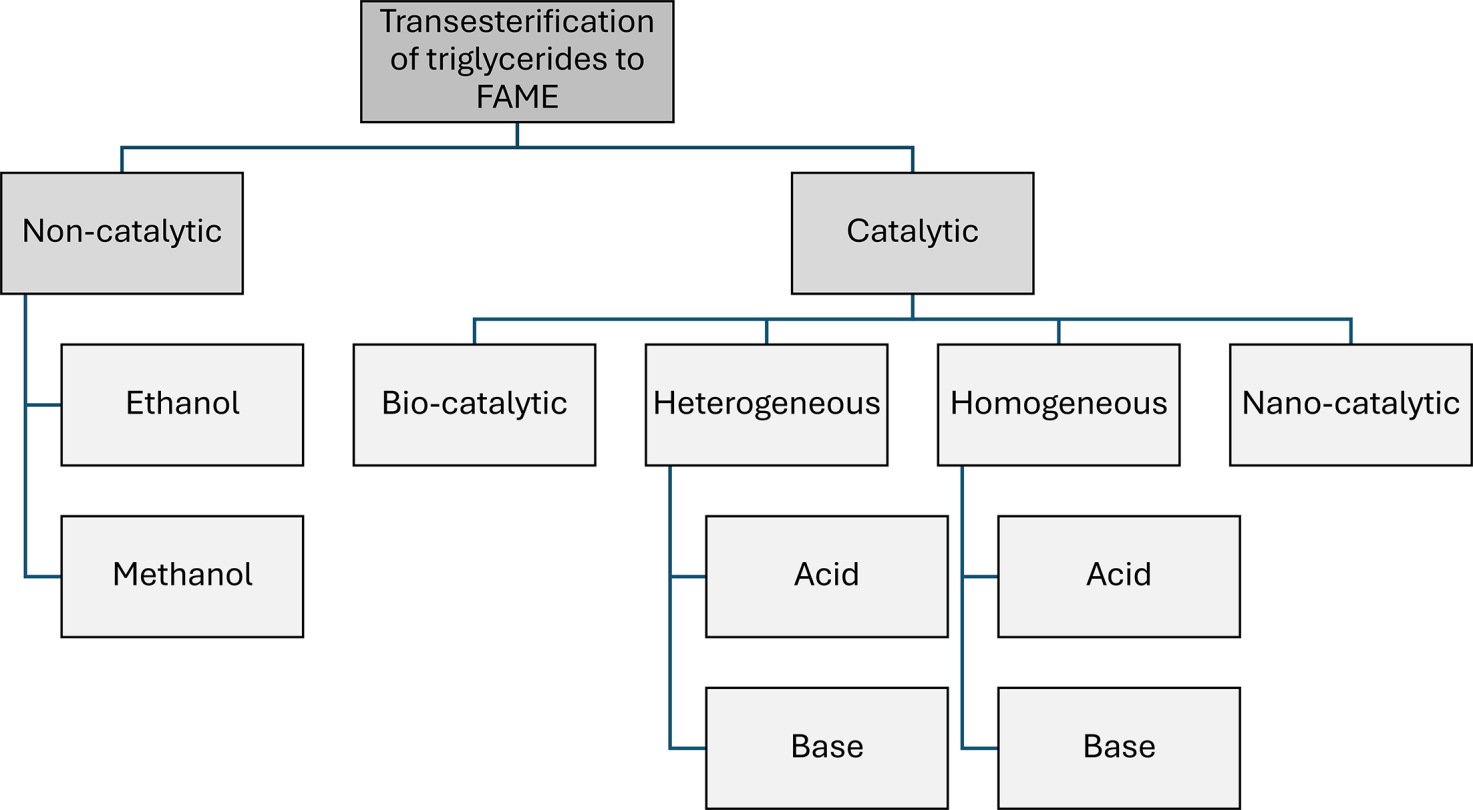
Conversion of lipids to biodiesel via transesterification processes, including non-catalytic methods and catalytic techniques (homogeneous, heterogeneous, biocatalytic and nanocatalytic).

The microalgae species *Chlorella vulgaris* has been extensively studied for its high lipid productivity and adaptability to diverse cultivation environments. The effectiveness of *in-situ* transesterification under subcritical conditions was demonstrated, achieving a 74.6% FAME yield at 220°C using biomass with 80 wt% moisture content [53]. This process optimised methanol usage to 8 mL per gram of biomass and consumed 0.47 kWh of power, showcasing its energy efficiency relative to traditional methods. Integrating lipid extraction with transesterification eliminated intermediate drying steps, significantly reducing overall processing costs. Such advancements underscore the importance of process optimisation to achieve scalable and sustainable biodiesel production from *Chlorella vulgaris*. These findings also highlight the potential of combining extraction and conversion steps into a single streamlined process, minimising energy consumption and material waste (Figure 4) [54].

Catalytic transesterification processes, classified into homogeneous, heterogeneous, biocatalytic and nanocatalytic methods, use catalysts to improve reaction rates and biodiesel yields [52,55]. Homogeneous catalysts include alkali catalysts like sodium hydroxide and acid catalysts like sulphuric acid. While alkali catalysts provide high conversion rates under mild conditions, acid catalysts are more effective for feedstocks with high free fatty acid content [50,56–61]. Heterogeneous catalysts are solid catalysts such as calcium oxide (CaO) and magnesium oxide (MgO) that offer reusability and tolerance to free fatty acids. However, they may experience leaching and reduced stability over time. These catalysts are particularly effective in continuous production systems [62–64].

Enzymes are protein-based biocatalysts derived from tissues, plants and microorganisms such as yeast, microbes and fungi. They are highly selective, efficient and eco-friendly, and they find applications across industries, including biofuel production, dairy, cooking, detergents, leather, paper and textiles [34,55].

Advances in enzyme immobilisation have addressed many challenges associated with enzymatic instability, particularly in industrial-scale applications. Techniques such as immobilising enzymes on nanoparticles significantly enhance their stability by increasing surface area and improving enzyme–substrate interactions. For instance, lipases immobilised on magnetite nanoparticles have demonstrated higher catalytic efficiency, thermal stability and reusability than free enzymes [12,65,66]. Cross-linked enzyme aggregates and microwave-assisted immobilisation methods enhance enzyme activity by stabilising their structure and preventing denaturation under extreme conditions.

Additionally, using ionic liquids (ILs) as co-catalysts with immobilised enzymes improves mass transfer efficiency while reducing the toxicity associated with organic solvents. ILs have been shown to increase biodiesel yields by facilitating substrate–solvent interactions, with hydrophobic ILs yielding superior performance [67]. For example, *Candida antarctica* lipase B immobilised with 1-hexadecyl-3-methylimidazolium bis(trifluoromethylsulfonyl)imide IL exhibited a catalytic activity of 245.13 U/g, significantly surpassing conventional solvent systems [68]. These advancements make enzymatic biodiesel production a promising eco-friendly alternative to traditional methods, with ongoing research to further reduce material costs and improve large-scale applicability.

Nanotechnology improves reaction efficiency through materials with high surface areas and reactivity [69,70]. Nanocatalysts mitigate saponification, providing a scalable alternative to conventional catalysts [71]. Their use in biodiesel production has demonstrated enhanced yields and reduced energy consumption [70–72].

The effectiveness of different transesterification methods, including their associated biodiesel yields, is summarised in Table 2.

**Table 2:** Biodiesel yields from different transesterification processes of microalgal oils (laboratory and pilot-scale data).

Process	Algal species	Catalyst	Solvent	Biodiesel yield (%)	Ref.
**Non-catalytic**	*Chlorella protothecoides*	-	Supercritical methanol	95.5	[73]
	*Schizochytrium limacinum*	-	Supercritical dimethyl carbonate	50	[74]
	*Nannochloris sp*	-	Supercritical methanol	21.8	[75]
	*Spirulina sp*.	-	Supercritical methanol	72	[76]
	*Chlorella protothecoides*	-	Supercritical ethanol	88	[77]
**Homogeneous catalytic**	*Neochloris oleoabundans*	NaOH	Methanol	83.8	[78]
	*Chlorella sp. BDUG 91771*	H_2_SO_4_	Methanol	60	[79]
	*Chlorella vulgaris*	KOH	Methanol	90.4	[80]
	*Nannochloropsis gaditana*	HCl in methanol/chloroform	Methanol	>90	[81]
**Heterogeneous catalytic**	*Chlorella vulgaris*	KOH/Al_2_O_3_	Methanol	89.5	[82]
	*Chlorella protothecoides*	CaO/dolomite	Methanol	>90	[83]
	*Scenedesmus quadricauda*	Cobalt-doped CaO	Methanol	98	[84]
	*Scenedesmus obliquus*	WO_3_/ZrO_2_	Methanol	94.6	[15]
	*Nannochloropsis oculata*	CaMgO/Al_2_O_3_	Methanol	85.3	[85]
	*Scenedesmus obliquus*	Cr-Al mixed oxide	Methanol	98.3	[86]
**Biocatalytic**	*Chlorella protothecoides*	Immobilised lipase Candida sp. 99-125	Methanol/water	98.2	[87]
	*Neochloris oleoabundans*	Plant-based ferric oxide	-	86	[88]
	*Chlorella pyrenoidosa*	[BMIM][PF_6_]/*Penicillium expansum* lipase CAL	-	90.7	[89]
	*Chlorella vulgaris*	[C_16_mim][NTf_2_] and [Bmim][Cl]/CAL B	-	100	[90]
	*Nitzschia punctata*	*Cladosporium tenuissimum* lipase	-	87.2	[91]
**Nanocatalytic**	*C. vulgaris ESP-31*	*Burkholderia sp*. C20, immobilised on nanocomposite Fe_3_O_4_–SiO_2_	Methanol/hexane	95.1	[92]
	*Tetraselmis indica*	Nano-Ca(OCH_3_)_2_	-	99	[71]
	*Tetraselmis indica*	Nanocatalyst LIICO (lithium-ion–impregnated CaO)	-	93	[71]
	*Chlorella vulgaris*	Immobilised *Rhizopus oryzae* lipase (ROL) on superparamagnetic iron NP	-	68.8	[93]
	*Chlorella vulgaris*	Nano-CaO	Methanol	-	[94]
	*Neochloris oleoabundans*	Nano-Fe_2_O_3_	Methanol	86	[70]
	*Spirulina sp*.	Nano-Ca(OCH_3_)_2_	Methanol	99	[95]
	*Ulva lactuca*	Si/ZnO	Methanol	97.3	[96]

## Emerging challenges and opportunities

While these methods have advanced biodiesel production, challenges such as high energy consumption, catalyst deactivation and process scalability remain [97]. Future research should prioritise integrating these technologies into cost-effective and environmentally sustainable systems to address these constraints.

### Challenges and future prospects

The global reliance on fossil fuels underscores the urgency of transitioning to sustainable energy sources that align with existing infrastructure. Biodiesel, as a renewable transport biofuel, offers compatibility with current systems and a significantly reduced carbon footprint, making it a promising alternative to conventional diesel [97]. However, large-scale adoption of microalgal biodiesel faces critical challenges that require innovative solutions [98,99].

### Life cycle assessment and sustainability metrics

Life cycle analysis plays a pivotal role in assessing the environmental impact of algal biofuels [100]. Key parameters include the NER, greenhouse gas emissions, water usage and waste generation. Microalgal biodiesel currently achieves an NER of approximately 2.5, which is lower than the NER of 5 for fossil diesel. This gap highlights the importance of energy-efficient cultivation, harvesting and processing techniques to improve sustainability and economic feasibility [101].

### The role of nanotechnology

Nanotechnology offers innovative solutions to overcome technical and economic barriers in microalgae-based biodiesel production. Nanocatalysts, such as nano-CaO derived from low-cost materials, exhibit high reusability, enhanced reaction rates and biodiesel yields exceeding 99%. By improving these processes, nanotechnology supports sustainability targets identified in life cycle assessment studies and cost reduction goals, positioning it as a critical enabler for commercialisation [100].

### Strategies for commercialisation

Future strategies must focus on integrating microalgae biorefineries with other industries to achieve commercial viability. Co-extraction of high-value bioproducts, such as proteins, pigments and bioplastics, can enhance profitability while supporting a circular bioeconomy. Additional measures include leveraging rural areas for low-cost cultivation, integrating aquaculture to create synergies, and using wastewater as a nutrient source to lower operational costs and reduce environmental impact [102]. For example, integrated aquaculture–algae systems in Asia (e.g. shrimp farms in Thailand) have successfully applied nutrient-rich effluent to algal growth, demonstrating how nutrient recycling can lower feed costs and improve water quality [103,104].

Despite significant laboratory progress, large-scale deployment of microalgal biodiesel remains constrained by cost and scalability barriers. Techno-economic assessments estimate current production costs at approximately US$4–6 per litre, driven by energy demands for cultivation, harvesting and lipid extraction [105]. Recent pilot-scale studies integrating wastewater-based cultivation, nutrient recycling and automated PBR controls have demonstrated potential cost reductions in 20–30% [106]. These insights directly connect technological innovation to industrial feasibility, underscoring the need for aligned research and policy frameworks.

### Carbon trading and economic incentives

The ability of microalgae to sequester carbon dioxide and produce secondary products positions them as an attractive option for carbon trading. High-value bioproducts, including fatty acids, vitamins and pigments, offer economic incentives for energy companies, potentially accelerating investments in algae-based biofuels [107–110]. For instance, Algenol biofuels (Florida, U.S.A.) demonstrated CO₂ utilisation by recycling industrial emissions into algal cultivation, illustrating how such systems could integrate with carbon credit frameworks to incentivise large-scale adoption [111]. In addition, the US EPA recognised Algenol’s platform for integrating CO₂ utilisation with co-production of valuable pigments and biofuels, providing a model for scalable biorefineries [104].

### Research priorities

Future research must prioritise innovative farming techniques, such as automated and precision-based cultivation, to optimise productivity and scalability. Developing genetically modified strains tailored for industrial-scale production and metabolic pathway optimisation can further boost lipid yields and resource efficiency. Moreover, emphasis on reducing energy inputs across all stages, from cultivation to conversion, remains essential to align with life cycle targets and ensure economic feasibility [112].

Recent AI and machine learning advances are accelerating microalgal strain development and process optimisation. Machine learning models have achieved predictive accuracies exceeding 90% for lipid productivity by integrating cultivation parameters such as nutrient levels, light intensity and pH [113]. A random forest approach has demonstrated 95.2% accuracy in optimising lipid productivity in *Chlorella* sp. by balancing biomass productivity and lipid content, identifying iron and phosphorus availability as key drivers over traditional nitrogen starvation strategies [114]. Similarly, deep learning and hybrid algorithms have been applied to optimise PBR conditions, enabling *in silico* fine-tuning of CO₂ flux, illumination and mixing regimes before experimental validation, improving lipid yields while reducing operational costs [115]. AI-driven metabolic modelling has also highlighted gene targets for CRISPR-based enhancement, reducing iterative experimentation. By integrating AI with omics data and automated high-throughput screening, these approaches offer a scalable framework to shorten development timelines, improve resource efficiency and bridge the gap between laboratory innovation and industrial implementation [113,115].

In summary, addressing these challenges through interdisciplinary research, technological innovation and policy support will be crucial for realising the economic and environmental benefits of microalgae-based biodiesel. With continued progress, microalgae biorefineries have the potential to play a transformative role in the global energy transition.

**Summary**

- Microalgae as a sustainable biodiesel feedstock: microalgae offer high lipid productivity, rapid growth and the ability to grow in non-arable land and wastewater, positioning them as a cornerstone of third- and fourth-generation biofuels.
- Advances in technology: recent developments in genetic engineering, nanotechnology, AI-driven strain optimisation and hybrid cultivation systems have significantly improved biodiesel yields and process efficiency.
- Integration into biorefineries: co-production of high-value bioproducts (pigments, proteins and nutraceuticals) enhances economic viability and supports circular bioeconomy models.
- Remaining challenges: high energy costs, scalability constraints and limited industrial deployment remain barriers; life cycle assessments (net energy ratio ~2.5) highlight areas for improvement.
- Future directions: to transition microalgal biodiesel from laboratory to commercial reality, it is essential to focus on techno-economic optimisation, pilot-scale demonstrations and supportive policy frameworks.

## Conclusion

Microalgae hold immense promise as a sustainable feedstock for biodiesel production, addressing both environmental and energy challenges. Their ability to thrive in diverse environments, including non-arable land and wastewater, with high lipid productivity and rapid growth, positions them as a leading candidate for third- and fourth-generation biodiesel, reducing dependence on fossil fuels and mitigating climate change impacts.

Future work should prioritise pilot-scale demonstrations that validate laboratory innovations under industrial conditions, including automated PBR controls and wastewater-integrated cultivation systems to reduce costs. Research into strain engineering via CRISPR-Cas9 and AI-driven process optimisation should be coupled with techno-economic modelling to guide scale-up. By integrating AI, CRISPR and nanotechnology with sustainability insights, microalgal biodiesel can advance beyond laboratory constraints toward commercial viability.

Policy measures, such as carbon credit incentives for CO₂ biofixation, feed-in tariffs for algae-derived fuels and nutrient recycling regulations, alongside collaboration between academia, industry and government, will be critical to aligning research breakthroughs with infrastructure and market development. The co-production of high-value bioproducts also strengthens the feasibility of microalgae biorefineries within a circular bioeconomy.

Despite decades of research, algal biodiesel remains commercially unrealised. Production costs (US$4–6/l) exceed fossil diesel pricing, NERs remain suboptimal (NER ~2.5 vs. ~5 for fossil diesel), and large-scale cultivation faces contamination and infrastructure challenges. Highly variable life cycle assessments further limit definitive sustainability claims. As a result, most efforts have shifted toward high-value co-products rather than bulk fuel. Demonstrating cost competitiveness and sustainability will require integrated techno-economic optimisation, validated pilot-scale data and targeted policy support to close the gap between laboratory advances and industrial feasibility.

## Open Access

This article has been published open access under our Subscribe to Open programme, made possible through the support of our subscribing institutions, learn more here: https://portlandpress.com/pages/open_access_options_and_prices#conditional

## Abbreviations

AI: artificial intelligence
CaO: calcium oxide
FAMEs: fatty acid methyl esters
GEM: genetically engineered microalgae
ILs: ionic liquids
NER: net energy ratio
PBR: photobioreactor
